# Stimulation *vs.* Depletion

**Published:** 1861-01

**Authors:** Patrick Fraser

**Affiliations:** Physician to the London Hospital


### MISCELLANEOUS.

### STIMULATION *VERSUS* DEPLETION.

BY PATRICK FRASER, M. D.,

*Physician to the London Hospital.*

The question as to the utility or the non-utility of venesection in the treatment of disease, has engaged the attention of men from the earliest stages. Much has been written upon it, conflicting opinions offered, and unsatisfactory conclusions drawn; but it is reserved to the observer of the present century to witness the great contest on this matter.

The fact cannot be ignored that a remarkable change in the treatment of those alterations in the human frame from a state of health which require the employment of stimulative, and the avoidance of depletive, measures, prevails in the profession. The difficulty is to reconcile the very discordant opinions expressed as to the causes for this change, and their operative extent.

One party asserts that venesection never was, and is not now, necessary, but positively injurious. Another party holds that venesection is now, as formerly, highly beneficial in many cases of disease; although it is now less requisite than formerly. Another, or third party, cuts the Gordian knot, and resolves the whole difficulty into a change of fashion in physic.

One might imagine, judging from recent writers, that this change began about the year 1815, and was perfected about 1833, but that vacillations of opinion on this very subject have been expressed long ago, and that our ancestors have in this, as they have in almost every other matter, foreshadowed changes, will be borne out in the sequel.

The fashion party may be dismissed by observing that the allegation as to the frequent changes, do not apply in the present matter. The fashionable changes—admitting the term—are in mere matter of detail, not of principle, and refer to the substitution of one drug for another; but it is to be remembered that the new remedy has always more or less affinity to the one discarded, and has a similar or approximative action on the animal tissues.

The disputants may now be divided into two parties. The change is attributed by one party to our increased knowledge in the application of physical diagnosis; by the other, to a progressive alteration in the type of disease, or, as Sydenham has called it, "constitutio morborum stationaria."

I am induced to write this paper in order to place before the profession certain tables derived from the statistics of the London Hospital, the results bearing upon some very remarkable questions. But, before doing so, I think that it may not be uninteresting to many readers to give a brief history of the various opinions held upon venesection from the earliest ages, and for the further guidance of future investigators.

I may say, that having consulted nearly one hundred ancient and modern authors, I was suddenly checked in my (perhaps hopeless) labor by the statement of "Dr. Carol. Fred. Nopitisch," a physician of Nuremberg, who cites thirty writers on bleeding before the Christian era, and no less than seven hundred and ten from that period to the year 1830; and we pretty well know that the number is not small since. With this statement before me, I closed my portfolio in dismay, and determined to try something in the way of the Baconian rule, trusting that by a collation of facts, more than by theory, to arrive at some result.

First Epoch.—*From the earliest ages down to the time of Hippocrates.*

Bleeding was practised by the ancients under various impressions, such as that its effects were evacuant, revulsant, derivative, alterative, excitant, depressant. Prolix disquisitions on these various supposed powers prevail in their writings, and the fact of these opposite powers having been attributed to venesection exerts no small influence on the practice of medicine in the present day.

Our information as to the origin of bloodletting is imperfect, owing to the dark traditions of the ancients.

The Egyptians disapproved of bleeding, but the following is the version by that people of its rise: It was observed that when the hippopotamus had a headache, (whether after an undue potation is not stated,) he emerged from his watery bed, and performed the operation of bleeding by striking his ponderous foot upon some convenient sharp stone, thereby causing a flow of blood, which he arrested at pleasure by a pledget of mud. This interesting animal has thereby the credit of having been the earliest surgical operator, as well as having taught mankind the value and use of styptics.

Pliny (His. Nat., viii., 25, 26), in a more elegant fiction, states: "The sea-horse, when too full of blood, scratches himself on rose-bushes, and then staunches the flowing of the blood by rolling in the mud." Our rose-bushes must have deteriorated in their strength of prickles, for very few of the present day would much affect the hide of the modern hippopotamus. But however this may be, it is clearly recorded, like all other matters of ancient history, that Podilarius, one of the sons of Æsculapius, practised venesection, A. C. 1134, and that the first bleeding was made on the daughter of King Dametus. The fee was liberal, being not only the patient in marriage, (who we are bound to believe was both young and beautiful,) but the Chersonese to boot for a dowry.

From the earliest period of our race, the nasal and catamenial fluxes would be observed, and noticed as often beneficial; and down to the present time there have been practitioners who have taken spontaneous bleedings as indicative for the need for general depletion. We are therefore justified in concluding that artificial means of relief would be resorted to—if not, like the polished Tahitians, by opening a vein, at least, like the rougher natives of Guinea, by plunging a knife into the flesh at hap-hazard.

Arriving at more authentic periods, we know that Pythagoras (B. C. 580) disapproved of bleeding; that Hippocrates (B. C. 420) speaks of it as a practice well known, and that he himself practised it; for the name Anaxion is given as that of one of his patients who was bled on the eighth day of pleurisy. This being the only recorded case in which bleeding was practised by Hippocrates, is by many persons considered a proof that he did not often bleed. This conclusion is not supported, however, in other parts of his writings, for this truly wonderful man gives repeated and careful directions as to the veins to be opened, and the manner of the operation; and he gives a specific recommendation to practise it in cases of apoplexy, pneumonia, and pleuritis; and also advises that the bleeding from wounds, except those entering the "ventrem internum," should not be suddenly arrested, as by its flowing inflammatory action might be lessened.

In Sprengell's translation of the "Aphorisms," Sec. 10, Aph. 29, we have the ancient oracle speaking thus: "But if a violent fever should require bleeding, to do it in its full force and height would be to kill the patient; we must stay therefore, till the fever abates, and not bleed so much at once, but repeat it rather." This axiom is repeated by Celsus. At Sect. 10, Aph. 23, he again speaks: "Letting blood by the opening of a vein is no new thing; but to do this upon account of every disease is entirely new." Verily the oracle spoke then as would truly now apply.

SECOND EPOCH.—*From Hippocrates to Paracelsus.*

In the work of M. Freteau on "Bloodletting," there is a brief account of the successors of Hippocrates, of which, with some alterations and additions, we now give an abstract.

The most distinguished disciples of Hippocrates, namely, Diocles and Praxagoras (B. C. 340)—practised venesection; while Chrysippus and Erasistratus denounce the practice; and shortly afterwards Cleophantes employed wine extensively as a remedial agent. We may suppose, therefore, that at this remote period the discussion began, and has continued with varying fervor to our own time, as to the respective merits of depletion and stimulation in the treatment of disease.

The successors of these physicians departed into the different countries under the Roman government, and disseminated and supported the doctrines of their masters according to their adherence. For instance, Aselepiades (B. C. 90) rejected all evacuant remedies, especially bleeding; while Themison approved of its moderate use. This latter passes as the first physician who employed leeches, although there is mention made of their use so early as B. C. 140. Celsus (A. D. 10) bled children, old men, and pregnant women. Aretæus (A. D. 90) speaks of venesection, leeching and blistering. Galen (A. D. 131) declared himself an adherent to the principles of Hippocrates, and bled frequently; his chief indications were to evacuate the redundant blood in a plethora, or as a revulsant; and he appears to be the earliest writer to specify the exact quantity of blood to be extracted in special cases of disease; and in certain cases he bled to the extent of inducing "deliquum animi." About the year 280, Severus, Antyllus, and Apollonius practised arteriotomy and scarifications. The successors of Galen pursued his mode of treatment. For example, Orabasius, in 360; Ætius, 540; Alexander of Tralles, in 570; Paul of Ægina, in 670; Mesne, Serapion, Rhazes and Avicenna, during the ninth and tenth centuries. The latter states that the usual quantity of blood in a man's body is 25

pounds, and that a man may lose 20 ℔s. at the nose, "and not die." Was this ever proved? Avenzoar, Averrhoes, and Albucasis, in the twelfth century; and the doctrines of the schools of Salernum, A. D. 1000; of Montpellier, A. D. 1200; of Paris, A. D. 1300; also, Gui de Cauliac, were Galenistic. The sect of Chemists appeared A. D. 1400, and their influence rather retarded the progress of the healing art, and the act of bleeding was reprobated. Further information upon the opinions of the ancients on venesection, will be found in an excellent commentary in Adams's "Paulus Ægineta," p. 319.

In the works of all the men whose names have been mentioned, although bleeding is more or less recommended, then, as now, long and angry disputes occurred as to the prophylactic and curative powers of bleeding, as well as to what veins or arteries should be opened in certain diseases. One and all, however, agree in the necessity for care in the adoption of venesection, and that so powerful a remedy should be employed only "when the disease is of a strong nature, the patient in the vigor of life, and the strength unexhausted." Celsus goes so far as to say, that "to bleed a man in the first stage of fever is to kill him;" and Galen takes the precaution to give lists of diseases in which venesection should never be practised, as follows: cacochymia, plague, dysentery, low fever, intermittent fever, serous apoplexy, dislocations; and in those in which it should be only rarely practised—viz., fractures, renal calculi, small-pox, measles, inflammatory fever; but he recommends it strongly in sanguineous apoplexy, phrenitis, and cerebritis; and we shall see that the moderns have not much more to say on the matter.

### Third Epoch.—*From Paracelsus to Harvey.*

In the fifteenth century, Paracelsus commenced a reformation in medicine—a man who, "with all his faults," did good service as a thorough reformer to the science of Physic. It is quite certain that a man of his temperament would not stop short in the employment of any remedy promising to effect sharp and speedy cures. We consequently find that he practised venesection: but under what, if any, restrictions, we have no information. A reaction now took place, and Botelli, in 1577, advocated excessive bleeding, to the extent of two or three pounds at one operation, and this repeated four or five times at short intervals.

The cases on record of the large quantities of blood which have been lost without apparent injury, would lead to the conclusion, that the average quantity of blood in the body—viz., 30 ℔s. in the adult, has been under-estimated: for example, "A phlegmatic man lost by the nose, mouth, and urine, 18℔s. of blood, and yet there remained so much in him, that on the application of cupping-glasses, they were instantly filled with blood." (Schenck, Obs. Med., i., p. 172.) Brapavolus had a lady under treatment, in whom twenty-two pounds of blood passed from the nose; she recovered by the use of several remedies "one whereof was phlebotomy." (Comment. ad Aphor., xxiii., 15.) Marcellus Donatus (C. xxiii.) gives a case of eighteen pounds of blood issuing from the nose. Amatus Lusitanus relates a case where, within five days, twenty pounds of blood passed from the nose; another, forty pounds in six days, "whom he yet cured by phlebotomy." Montanus says he "cured one of the 'emeroids,' which bled every day for forty-five days, two pounds of blood and more." (Schenck, Obs., Med., xiii, p. 342.) Anculanus gives a case of twenty-five pounds of blood being lost in three days from a wound, and recovered; and several others are related, in which six and twelve pounds of blood where voided by the nose in a few hours, or days, but recovery always took place. Riolan has taken, in twelve hours, five pounds of blood without injury. He estimated that half the quantity of the blood in the body might be extracted without danger; and as he considered the whole quantity of blood varies in different nations, he arrived at the estimate, that a German may loose 15 ℔s.; a Frenchman, ten pounds; Spaniards, Moors, and Asiatics, less in the order of their standing. That Botelli had supporters in the practice of excessive depletion, we know from Asteus, a writer in 1583, who argued that ancient as well as modern physicians erred in bleeding too little rather than too much. Van Helmont, at the same period, although he did not denounce venesection, exposed the evils arising from excessive depletion. Although we are not prepared to subscribe to his celebrated motto, "Ego sane nemini sanguinem mitto," still it is a fair question whether the excessive bleedings enjoined by Botelli and his followers, were not more injurious than the total rejection of venesection by Van Helmont and his followers. Various writers—for example, Almarico, Blondelo in 1620, J. F. Buchone in 1629, Stahl, Thouvenal, Martianus, Lancisi, Baglavi, Pascoli, Forestus, Peater, Hoffman, Amatus, and, last, Victoris Trancavellus, supported more or less the doctrines of Van Helmont; but to the credit of most of these men, their condemnation of bleeding did not extend to the utter exclusion of it as a remedial agent, but to the avoidance of its abuse only. The last named writer enters more minutely in his condemnation by designating the diseases in which venesection ought not to be practised—viz., delirium, epilepsy, paralysis, catarrh, ophthalmia, nephritis, hypochondriasis, continued fever, scirrhus uteri, suppressio mensi, menorrhagia, ending by saying, "Nos non debere nimis proclivis nimisque liberales esse in detrahendo sanguine, ne dum Scyllam fugimus in Charybdem incidamus."

We see from the foregoing, that bleeding has been employed as a remedial agent from the earliest period of the history of the human family, but upon the most opposite and sometimes illogical principles.

FOURTH EPOCH.—*From Harvey to the Middle of the Eighteenth century.*

We now arrive at the important period when Harvey, in 1619, first publicly taught the doctrine of the circulation of the blood. It might be expected that upon this grand discovery, the wavering opinions as to the practice of bleeding would have become fixed and certain; but this consummation, "most devoutly to be wished," has not been realized; the same discrepancy of opinions and vacillations in practice are manifested in the writers of the period now under consideration. Unable, apparently, to determine on the real question whether, in certain diseases venesection should or should not be performed, the writers now busied themselves in angry discussions as to which vein should be opened in certain diseases, such as veins of the arm, hand, ankle, foot, tongue, nose, canthii majores, temple; in fact, each part of the body had some one to assert its priority. Matthæus Curtius, in the fourteenth century, enters fully into this question; and about 1534 several writers gave opinions very learnedly on the subject. For example: Leonardi Fuchsii—"Apologia contra Hieremiam Thrinerum de Venesectione in Pleuritide"—taught that the vein of the affected side ought to be opened; whereas Andræ Vaesalii at the same period affirmed just the contrary; and it is not until the year 1741 that M. Martin, in his treatise, gives such instructions as may be followed with safety and propriety at the present day. He tells us that bleeding is indicated when the patient is in the flower of his age, the strength unimpaired, the complexion good, the pulse equable, full, and strong; if he has had previously good nourishment, and if there is general heat of surface and no organic disease: that it is contra-indicated when the patient is enfeebled and the constitution not strong, the skin and extremities cold, the pulse feeble, soft, and intermittent; if the patient has led a laborious and badly nourished life; and if there should be organic disease. Sebastiano Bado, in 1663, asserted the absolute necessity for venesection in small-pox, and the other exanthemata. Also, M. Patin, a famous physician of Paris, writes in the year 1680, "There is no remedy in the world which does more miracles than bleeding." On the other hand, we find Ramazzina, Richter, and others strongly condemning it.

At the same period, equal differences of opinion prevailed amongst our own countrymen. Sydenham (A. D. 1666) says: "Primum in curatione locum phlebotomiæ attribuo, quæ sine salutis periculo hic omitte nequit." Heberden says: "Febres ardentes raro sanantur sine detractione sanguinis copiosa et repetita." We have a treatise in 1685 by Joannis Franciscus de Francisco, "De Hodierno Practicantium abusu Sanguinem Mittendi semper in Febribus." There is also a most elaborate treatise by Henry Stubbe, a physician in Warwick in 1671. He defends the practice of phlebotomy against the "audacious and impertinent strictures" of "one G. Thomson," contemptuously called "Bacon-faced," a "pseudo-chemist," and a "pretended disciple of my Lord Verulam." This said G. Thompson seems to have had certain strong and not always incorrect opinions upon his subject. He tells us that "We are taught by Divine Writ that in the blood—the *spiritus rubeus*—is life;" that "one would think that it should put a stop to their prodigal, profuse bleedings, if they did but consider with what difficulty nature brings this *solar liquor* to perfection." He was no fool when he observed, "They should never attempt—yea, rather abhor—to enervate in the least, by the lancet, the strength with its correlative blood and spirits, without which there is no hope of a cure; for," he adds, "manente causa, manet effectus." That Dr. Stubbe was honest in his belief as to the efficacy of venesection is fully evinced by his "practising what he preaches." He tells us: "In short, I have myself been let blood above fourscore times, and yet am lean; and so far from being feverishly inclined, that I never had any, except the measles once, and small-pox twice, and twice a tertian ague." Rejoice, ye physicians of the present time, at your exceptions!

An array of learned theses during this epoch, and closing in upon the end of the century, upon this universal subject,—"De Recta Sanguinis Missione,"—sufficiently attest the divided spirit of the time: Dr. White, in 1712; John Van Coxie, 1725; Dr. Mentyles, 1744; John Huggan, 1771; Francis Cuel, 1775; William Drannan, 1778.

FIFTH EPOCH.—*From* 1750 *to the present time.*

It is manifest that our forefathers were fully alive to the difficulties involved in the question of the employment of venesection in disease, also to the necessity for care and discrimination in its application; that they held in view the age, sex, temperament, condition of patient, climate, and prevailing diseases; that they were, as we are, often sorely puzzled by discordant opinions; and it is painfully evident that in ancient, as in modern, times blood has been let when it had better not, and not let when it had better been let. Clutterbuck felt this strongly, and he, not very logically, comes to the conclusion that bleeding is useful when it is useful, and hurtful when it is hurtful: somewhat like the justice, who, in his summing-up in a doubtful case, consoled himself by remarking, "If I'm right I'm right, and if I'm wrong I'm wrong."

Several writers allege that the change from depletion to stimulation dates from the first invasion of the cholera morbus in 1830; but we have in evidence that it began much earlier —in fact, that similar discussions date from a very early period. Lind, in 1774, in his essay on the "Health of Seamen," p. 159, says that the physicians in the West Indies began to doubt the benefits of venesection. Hewson, in his original paper, (vol. lx., p. 406, "Philosophical Transactions,") doubts the propriety of bleeding. That Cullen had misgivings is obvious. In book i., par. 139 and 362, of his "Practice of Physic," he says—"Nothing is more evident than that bloodletting is one of the most powerful means of diminishing the activity of the whole body." . . . . "It is, however, to be attended to, that a greater evacuation than is necessary may occasion a slow recovery; may render the person more liable to a relapse, or may bring on other diseases." (par. 140.) "From all this it must appear that the employing bloodletting in certain fevers requires much discernment and skill, and is to be governed by the consideration of the following circumstances: —1st, the nature of the prevailing epidemic; 2nd, the nature of the remote cause; 3rd, the season and climate; 4th, the diathesis present; 5th, the period of the disease; 6th, the age, vigor, and plethoric state of the patient; 7th, the patient's former diseases and habits of living; 8th, the appearance of the blood drawn out; 9th, the effects of the bloodletting that may have been already practised." (par. 142.) It may be seen that Galen and Trancavellus had long before laid down similar rules.

M. Freteau, in 1816, says: "De nos jours, les constitutions sont moins fortes, les individus moins plethoriques, les maladies franchement inflammatories moins communes."

So early as 1817, Dr. Christison noticed a change in the treatment of fever; but especial attention was drawn to this subject by him in 1834. He writes thus: "Our patients ceased to sustain free venesection, a few ounces of blood bringing on faintness, and the constitution refusing to rally afterwards." The contrast of the foregoing observation with what he writes in his thesis in 1819, is most remarkable. He there describes the "pulse as being at 160, hard, and incompressible; the temperature of the body at 107°; and the blood spouting out with amazing force on opening a vein." At the same period of time, Dr. Macintosh, a man of great energy and originality, who had for many years advocated strongly the practice of of bleeding, especially in the cold stage of ague, writes, in vol. i., p. 472, of his "Practice of Physic," "within the last fifteen years I have seen several cases where considerable injury had been inflicted by very large bleedings."

In the year 1820, a stronger reaction party existed in the College of Edinburgh, as indicated by several theses of that date, in favor of venesection. The following are examples: Wm. Garland says: "Inter omnia remedia ad morbos varios sanandos, quibus corpus humanum obnoxium est, nullum ma jore effectu vel eventu feliciore quam sanguinis detractio adhibetum." John Barne (1821) says: "Medici omnibus sæculis missionem sanguinis remedium esse eximium arbitrati sunt." John Halkenston (1821): "Nostrorum temporum opiniones variæ sunt sed in universum medicorum animi magis de hoc remedio, quam de plerisque aliis consentiunt."

It was about this time that we have it also recorded by truthful and acute observing physicians,—for example, Gregory, Radcliffe, Alison, Watson, and others,—that they required in their own persons, for acute disorders, large depletions, which were followed, as attested by all them, with great and unmistakable relief to their sufferings. We have seen also, in our explorations of the writings of ancient as well as modern authors, that the employment of bloodletting, as a remedial agent, was not always in a fixed ratio. Unfortunately, the medical writers of the different periods simply state the fact, but have not left us information as to their opinions of the causes. That causes do exist seems certain, for surely the cessation in the employment of bloodletting has followed, and not preceded, the change.

We have now reached a period in which even more angry and censorious discussions took place than heretofore, between men whose opinions demand reverential, careful, and watchful attention. Dr. Alison and Dr. Bennett were the champions. Both agreed as to the fact of a change in treatment having taken place, but differed in opinion as to the cause or causes. The former attributed it to an alteration in the type of disease, thereby inferring some occult change in the human constitution; and if this be true, we may also truly infer that the excessive bleedings practised by our forefathers were probably quite right and necessary, thus rescuing their mode of practice from the unmerited obloquy which has been cast upon it by several writers. Dr. Bennett places the change to an increased knowledge in the art of diagnosis. We have no intention of entering minutely into the merits of the late controversy, but we would observe, that it appears to us that Dr. Alison, in allowing his antagonists to apply their arguments to pneumonia alone, weakened his ground; for if this improved diagnostic power be worth anything, it ought to apply to all diseases which is not attempted; but to all diseases the limitation in bloodletting applies. Again; we admit that through our increased diagnostic power, we are better warned not to bleed in pneumonia after consolidation of the lung has taken place. But this does not explain why even in the acute state of crepitation bloodletting is seldom resorted to now, but which would have been practised thirty years ago, *pleno rivo*, even in the absence of the assumed superior diagnostic power, and with great benefit to the sufferers; neither will it explain the advance in the use of stimulants *pari passu* with a decrease in the employment of depletants.

Drs. Christison, Alison, and others, have alleged the change in the type of disease to proceed from certain atmospheric influences. The former dwelt strongly upon the impoverished state of the poor population, evinced by the scorbutic diathesis, and the prevalence of cholera; but the fact that all ranks of the people, from the patrician to the peasant, have become subservient to the change, invalidates poverty as the main cause, and we know that the change had begun long before the modern cholera appeared. This party does not, as has been alleged, declare that the *modus operandi* of inflammatory action, or its effects, is changed; but simply that inflammation is not now attended by high arterial action.

It has not been, and probably never will be, proved what causes are in operation to effect the remarkable variations which have occurred in the symptoms of disease, and in our anxiety to appear to have some definite views, we are almost impelled to the *non causa pro causa*.

We can scarcely avoid the conclusion that certain causes are operating in cycles; that, in fact, the human family are subjected to periodic changes, impressing, as it may happen, a stenic or asthenic character, on the prevailing diseases. If this idea should be recognised as a fact, the extraordinary discrepancy of opinion as to the curative value of bloodletting would be reconciled and explained. That this change is not merely local, but pervades the length and breadth of the land, and that it has been recognized throughout Europe and America, is shown by the general intolerance of bleeding, not only in man, but amongst domestic animals, as proved by veterinary practitioners. In our experience we have witnessed exemplifications of this change. We remember cases similar to those commented upon by Stephens, Turnbull, Watson, Symonds, Hastings, Richardson, and others, where the injected eye, flushed face, burning heat of the skin, violent arterial throbbings; the incompressible, sharp, and wiry radial pulsation; urgent dyspnœa, a red tongue, intense headache, and other local pains, unattended with muscular debility, appeared to render bloodletting imperative. Contrast such cases with those, with few exceptions, now witnessed in town and country practice, in which there is little or no heat of skin, no arterial excitement, feeble and easily compressed radial pulsation, a white, loaded, and flabby tongue, a pearly-white conjunctiva, not much pain, but extreme prostration, urging to the employment of stimulants.

We have reason to believe that these changes in the type of disease occur sometimes at very short intervals. In China, during the month of February, 1830, an epidemic fever occurred amongst the English sailors, in which the tongue was white and loaded, no arterial excitement, and no pain. In the month of May following, a second epidemic appeared, during which the symptoms were—headache; pulse from 80 to 110, full, and bounding; tongue clean; severe epigastric pain. The following comment was made at the time: "We believe this fever to be of a different type from that witnessed in February;" and the question is put, "Is bleeding necessary in both types?"

The history of influenza will furnish many similar instances.

Everyone knows the variations which have occurred in the symptoms of this malady, requiring a complete change of treatment. We know, indeed, that from the earliest ages the symptoms and organic lesions have varied in similar epidemics. There have been variations noticed even in the same epidemic at different periods of its history.

---

In the foregoing paper, we have seen the small result after all the disputation; and although the grand fact that an extraordinary increase in the employment of stimulants, and disuse of depletants, is going on is generally recognised by the profession, it has not been demonstrated by undeniable statistics. This we shall proceed to do. The tables *in extenso* would occupy too much of the valuable space of this journal, but we consider that the following summary will be sufficient to enable the reader to draw inferences and deductions. Seeing that the bloodletting controversy is still open, and as in its settlement there are involved points of great practical importance, it behoves all practitioners to note carefully the effects of the stimulative or depletive system in the treatment of disease; for it becomes a practical as well as a scientific question to ascertain the difference, if any, in the mortality in the practice of medical men, who notoriously prescribe excessive, moderate, or no stimulation at all, or the reverse; for only in this way, in this particular matter, can error be detected and truth substantiated.

It becomes more imperative in us to urge a general statistical inquiry, seeing that, in this the first published document of the kind we are obliged to admit that the results are negative and contradictory; and it will be only by a collation of similar reports that our aspirations may be realized.

We admit that, before we can draw absolute truth from statistical data in the matter, we ought to have an equal number of cases—the age, sex, and previous habits—an equality in the intensity and duration of the symptoms; for it is an inseparable element of disease that in no two cases will the phenomena be exactly alike; and it is this very dissimilarity that calls for the acumen and observing power required by the medical practitioner. But it is evident that, if this rule were rigidly enforced, we should be deprived of all aid from medical statistics; and we must be content, therefore, with approximative truth.

Considering the importance of the question at issue, whether taken in a sanitary, prophylactic, or financial view, it would be highly valuable to possess authentic records as to the practice of venesection and other depletory measures, and of the expenditure of the various items comprised under tonics, stimulants, etc., with the corresponding mortality, in the various hospitals of London, and elsewhere. Unless this general information be possessed, a serious error would arise in an attempt to generalize upon limited data. We must take into account also the varying and unavoidable causes in constant operation, quite irrespective of any peculiarities in the mode of treatment and diet, such as good, bad, or indifferent drainage and ventilation, and all other sanitary conditions which must affect the mortality of a large general hospital.

In the following summary of the tables, we give, for the convenience of comparison, three decennial periods.

In 1835, there were treated as in-patients in the London Hospital, 2735 persons, of whom 277 died, being at the rate of ten per cent. During that year, there was expended for these 2735 patients, the sum of £615 for beer; £346 for wine; making a total of £961. To counterbalance the effect of these stimulants, blood was drawn by 33,950 leeches, to say nothing of bleeding and cupping, then largely practised.

In the year 1845, there were treated as in-patients 3625 persons, of whom 228 died, being at the rate of 6¼ per cent.—These patients consumed, in the course of their treatment, wine and spirits to the amount of £848; or, to be more minute, they drank 10 gallons of brandy, 236 gallons of wine, 10 gallons of gin, and 96 gallons of spirits of wine besides were exhausted. To meet this alcoholic accumulation, blood was drawn by 33,800 leeches, to say nothing of venesections, cuppings, etc.

But what transpired in 1855? In that year, 3947 patients were treated in the Hospital, of which number 294 died, being at the rate of 7½ per cent. This number of patients consumed during the year, 143 gallons of brandy, 1153 gallons of wine, 60 gallons of gin, and 144 gallons of spirits of wine were exhausted, at a cost of £1796, as against £848 and £961 for the two previous decennial periods. But now appears the strange fact, that during the year 1855 the effects of this accumulation of alcohol were diminished by the quantity of blood drawn by 4400 leeches only, with, perhaps, not a single venesection, and at an average of not more than two cuppings per week, as against 33,800 and 33,950 leeches, frequent venesections and cuppings, for the two previous decennial periods.

Pursuing this inquiry, it is found that in the year 1837 there were used in the London Hospital only 29 oz. of quinine, and 101 lbs. of bark. Whereas in the year 1857 the consumption of these and analogous drugs were as follows: 54 oz. of quinine, 478 lbs. of bark, 600 oz. of the disulphate of bark, and 47 oz. of the quinine with the citrate of iron. The number of patients in 1837 being 2961; in 1857, 3935; and the per-centage of deaths being respectively—for 1837, 14 per cent.; for 1857, 8 per cent.

The two years above noted are sufficiently approximate to allow the same line of argument as for 1835 and 1855.

Attention should be drawn to the fact that in the year 1844 there was expended for stimulants only £821, being the *smallest* amount during a period of 22 years; and that the mortality in that year was 6 per cent. the *smallest* per centage of mortality during the said period of 22 years. We are not quite satisfied but that, if a rigid inquiry were made, the largest mortality would be found where the largest quantity of stimulants was given. It is to be observed that in the year 1851, when the number of patients—viz., 4051—was larger than in 1857—viz., 3935,—the mortality was nevertheless greater during the latter year, as 8 per cent. to 6.50 per cent.; although the increase of expenditure for articles of luxury, &c., during 1857, exceeded that of 1851 by the large sum of £962.

Another puzzle is presented on looking upon the results of the year 1858. We have in this year a less consumption of wine and spirits (namely £573 3*s.* 3*d.*) than during the preceding 23 years, the average for the 24 years being £1055; but the quantity of tonics remains nearly the same as in 1855:—Ammonia sesqui., 140lbs.; quinine, 50 oz.; quinine and citrate of iron, 5lbs.; bark, 511lbs.; disulphate of bark, 660 oz.; leeches, 3900.

The table from which the following additional summary is compiled brings into view rather startling and puzzling facts. It extends over a period of ten years, and therefore is a perfect average, and we have divided it, for our present purpose, into two quinquennial periods. It shows a direct rising increase annually in the expenditure of wine in the treatment of their patients by the physicians and surgeons of the hospital; and it so happens that from changes in the staff this decennial period embraces the practice of six physicians, rendering the alterations in, at all events, the medical practice less likely to have arisen from any peculiar or personal views.

To economize space, we have not given the quantities employed by each individual physician or surgeon, but the aggregate.

From the year 1849 to 1853 inclusive, the annual average quantity of wine employed by each physician was 4928 ounces. During the same period the annual average number of patients under the treatment of each physician was 390; the annual average mortality being 9-68 per cent.

From the year 1854 to 1858 inclusive the annual average quantity of wine employed by each physician was 12803 ounces. During the same period the annual average number of patients under the treatment of each physician was 391; the annual average mortality being 11-87 per cent.

In the surgical wards a similar unexpected result is observed.

From the year 1849 to 1853 inclusive, the annual average quantity of wine employed by each of the surgeons was 17,533 ounces. During the same period the annual average number of patients under the treatment of each surgeon was 1075; the annual average mortality being 4-48 per cent.

From the year 1854 to 1858 inclusive, the annual average quantity of wine employed by each of the three surgeons was 38,016 ounces. During the same period the average annual number of patients under the treatment of each surgeon was 1036; the annual average mortality being 5.06 per cent.

It may be supposed that, as regards the medical cases, this unexpected result proceeded from a difference in the kind and severity of diseases which were fatal during the periods included in the calculations. Information on this point in these cases is not available; but as regards the surgical cases the record is complete and correct, and from it we learn that this cause does not operate, as the remarkable fact is demonstrated by another table that the annual average number of each kind of accident during a period of ten years is very nearly alike; thus adding one more illustration of the unexpected fact elicited by statistics—namely, that the mechanical causes producing accidents in a fixed population are pretty permanent; for we cannot assume that there has been an unusual severity in the character of the accidents. The table gives a more extended basis, as regards surgical cases, to this inquiry, as it includes the outpatients as well as the in-patients; and we see at once that the same regularity continues in the kind of accidents with the larger number of patients. We repeat that we should be very sorry even to attempt to generalize on the limited data now presented to the reader; nevertheless we do consider that the facts brought out in these tables are of sufficient weight and importance to warrant us in asserting that the profession ought to pause in an indiscriminate employment of stimulants and tonics, especially the former, in the treatment of disease, even of apparently an asthenic character. Many excellent men—Mr. Higginbottom, for example—have denounced the stimulant treatment *in toto.* We are not yet prepared to go so far, for where there is so much of doubt, it behoves all of us to entertain tolerant views. —*London Lancet.*

